# Ketamine as an alternative to ECT in catatonia in elderly women with bipolar disorder: A case report

**DOI:** 10.3389/fpsyt.2023.1138772

**Published:** 2023-04-13

**Authors:** Shanthi Sarma, Arulmathy Arunachalam, Memunatu Kamara, Grace Branjerdporn

**Affiliations:** Gold Coast Hospital and Health Service, Gold Coast, QLD, Australia

**Keywords:** ketamine, catatonia, COVID-19, electroconvulsive therapy (ECT), depression, bipolar disorder

## Abstract

The following paper described two cases of patients with catatonic depression in bipolar disorder (BD) referred to our electroconvulsive therapy (ECT) service. Both were deemed not medically fit for ECT, and were, instead, treated with intravenous (IV) ketamine. Both responded with a resolution of symptoms, returning to baseline level of functioning. During the COVID-19 pandemic, given the risks associated with providing ECT (an aerosol generating procedure) and, in the context of limited resources, ketamine therapy for catatonia is a potentially beneficial alternative or supportive treatment to ECT that merits additional research.

## 1. Introduction

Catatonia is a severe but treatable neuropsychiatric syndrome characterized by a number of distinctive psychomotor disturbances, including mutism, stupor, immobility, excessive motor activity, echolalia or echopraxia (1, 2). Currently, the gold standard of treatment for catatonia is electroconvulsive therapy (ECT), with an efficacy rate of 80 – 100% (2, 3).

The COVID-19 pandemic presented a number of new and unique challenges regarding infection control and safety when delivering ECT. Bag-mask ventilation during ECT produces aerosolization of respiratory secretions that pose a high risk of disease transmission during the outbreak of a contagious, airborne virus (4). Clinicians have attempted to overcome these issues in a number of ways; using adjusted PPE protocols, or delaying and reducing ECT treatments (5, 6). Patients may also be considered medically unfit to receive ECT, and alternative treatments for catatonia must be delivered.

In recent years, the fast-acting anesthetic, ketamine, has been given wider use in psychiatric pharmacology. Ketamine is an N-methyl-D-aspartate (NMDA) receptor antagonist, believed to work by acting on abnormal NMDA signaling and elevated glutamate often observed in major depressive disorder (MDD) and BD (7). A number of studies have found that low dose infusions (0.5 mg/kg) of ketamine significantly reduce depressive symptoms and suicidality in treatment resistant depression (8, 9). These effects have a rapid onset and are maintained for a number of days (10). Increased infusion rates have been observed to increase the response and duration of antidepressant activity (9).

Originally considered a catatonia-inducing drug, recent advances in knowledge around the role of glutamate and NMDA receptor activity in catatonia have pointed to ketamine as a promising treatment (11). However, few studies to date have investigated its use in catatonia. Only two case studies have been published that describe the use of ketamine in catatonic subjects; both presenting promising findings (11, 12). The current paper presents the case studies of two patients with bipolar disorder (BD) presenting with catatonic depression, describing their successful treatment with ketamine.

## 2. Case presentation

### 2.1. Case 1

#### 2.1.1. Chief complaints

The patient, an 81-year-old woman with BD, presented with depressive catatonia associated with stupor, mutism, and extreme negativism.

#### 2.1.2. History of present illness

Her family described a 3-month history of worsening mood, characterized by social withdrawal and anhedonia, spending most of her time in her room in bed. This occurred after the patientx decided to stop taking her medications. She reported difficulties falling asleep and early morning wakening, and would lie in bed worrying about how she would get through each day. This was associated with a decreased appetite resulting in a ten-kilogram weight loss. On admission, pressure areas were evident on her sacrum.

#### 2.1.3. Past Psychiatric History

She had a long history of BD first diagnosed at age 24 characterized by multiple depressive and several manic episodes, with good inter-episodic functioning, and previous response to ECT when depressed. Her last episode had been 5 years prior. Psychiatric medications were Venlafaxine 150 mg mane, Mirtazapine 30 mg nocte, Sodium Valproate 500 mg nocte, 200 mg mane and Olanzapine 10 mg nocte.

#### 2.1.4. Past medical history

Past medical history included diabetes mellitus type II, ischemic stroke, hyperlipidemia, and ischemic heart disease.

#### 2.1.5. Psycho-social history

Prior to the current episode, she enjoyed neighborhood strolls with an aid, socializing with family, and playing bingo. She had no cognitive impairment and lived in residential aged care due to poor mobility.

#### 2.1.6. Laboratory examinations

Admission investigations were unremarkable [neuroimaging revealed an old posterior right Middle Cerebral Artery (MCA) territory infarct, blood investigations were normal including thyroid function tests]. An echo performed revealed severe Hypertrophic Obstructive Cardiomyopathy with a left ventricular outflow tract pressure gradient of 99 mmHg, deeming her medically high-risk for ECT.

#### 2.1.7. Diagnostic assessment and treatment

The patient was admitted medically for rehydration and nasogastric (NG) feeding. Venlafaxine XR was increased up to 300 mg daily, Mirtazapine was increased to 60 mg *via* NG. An IV lorazepam challenge test, titrated up to 3 mg, was administered with no response. A cardiology review was conducted, and the patient was placed on a beta-adrenergic blocking agent. As an alternative treatment to ECT for her severe depression, trial of IV ketamine was commenced. Prior to commencement, Montgomery-Åsberg Depression Rating Scale (MADRS) was 50 and Bush Francis Catatonia Rating Scale (BFRS) was 18. Ketamine at a dose of 0.5 mg/kg (40 mg) administered IV over 40 min was delivered twice weekly.

#### 2.1.8. Outcome and follow-up

**FIGURE 1:**
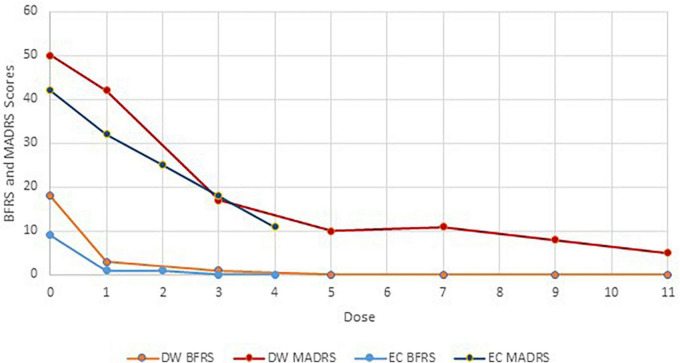
Montgomery-Åsberg Depression Rating Scale and BFRS scores and IV ketamine treatment course.

Within 2 h of first treatment, the patient started communicating verbally, expressing an improvement in mood. She received a total of 11 treatments (see Figure 1) and once improvement plateaued (MADRS = 5), ketamine ceased, and she continued on oral antidepressants. Her appetite returned, she expressed improvement in her mood and increased enjoyment -enjoying watching sports on television. She started to engage in physical rehabilitation. She was followed-up in the community for 6 months and remained euthymic and was able to be referred back to her general practitioner (GP) for ongoing management.

### 2.2. Case 2

#### 2.2.1. Chief complaints

The patient, a 76-year-old woman with BD, was admitted with catatonic depression, tachypnea, and low oxygen saturations (80–90% on room air).

#### 2.2.2. History of present illness

The depressive episode commenced 6 weeks prior, triggered by a course of oral steroids following surgical removal of cataracts. The patient started to become more anxious and tearful, losing confidence in mobilizing independently and became withdrawn, remaining in bed. She was no longer enjoying reading or speaking with her friends, and had become mute. There was neurovegetative disturbance with loss of 8 kilograms. She displayed negativism when attempts were made to examine her.

#### 2.2.3. Past psychiatric history

The patient had a long history of BD, characterized by brief manic episodes and long depressive episodes with catatonia, which had responded to ECT previously. She was cognitively intact and her inter-episodic functioning was good. The patient’s psychiatric medications were nortriptyline 50 mg nocte, lithium 250 mg bd, and temazepam 10 mg nocte.

#### 2.2.4. Past medical history

Past medical history included bronchiectasis, interstitial pulmonary fibrosis requiring a home oxygen concentrator, pulmonary embolus, hypertension, Gastroesophageal reflux disease, atrial fibrillation, and hyperlipidemia.

#### 2.2.5. Psycho-social history

The patient was a retired librarian and remained cognitively intact. She was independent with activities of Daily Living (ADLs), enjoyed social outings, and had supportive children.

#### 2.2.6. Further diagnostic work-up

Admission investigations revealed a small pulmonary embolus. Despite optimizing her medical condition, she required ongoing supplemental oxygen and NG feeding.

#### 2.2.7. Treatment

Despite an IV lorazepam challenge, there was no improvement in the catatonic symptoms and ECT was commenced. At her first ECT treatment, she desaturated significantly, resulting in critical saturation levels and deeming her high risk for further anesthetics. Due to the patient’s pulmonary history and intermittent home oxygen saturation fluctuating around 90%, treatment of the PE was considered unlikely to provide an adequate or timely response in order to recommence her ECT. Treatment of her catatonic depressive symptoms was the priority so a trial of ketamine was commenced. Prior to treatment, her MADRS was 42 and BFCRS was 9. Ketamine at a dose of 0.5 mg/kg was administered IV over 40 min twice weekly.

#### 2.2.8. Outcome and follow-up

There was a noticeable improvement following the first treatment, with a reduction in agitation, increased reactivity, and improvement in appetite (see Figure 1). Following her second treatment, the patient’s wry sense of humor returned, and she enjoyed spending time with her children and grandchildren. She described the treatments “surreal,” experiencing herself standing outside her body. Following her third treatment, she reported no further depressive symptoms, and requested to stop ketamine due to dissociative symptoms experienced during treatment. Her mood continued to improve over the next several days, with MADRS decreasing to 11. The patient was followed up in the community for over a year with no relapse of catatonic or depressive symptoms and was discharged from community psychiatric services.

## 3. Discussion

The use of ketamine for depression (both unipolar and bipolar) is rapidly expanding. Ketamine is a rapid-acting anesthetic that disrupts association pathways in the brain causing dissociative anesthesia (13). Clinical studies have demonstrated that a single sub-anesthetic dose of ketamine induces rapid and sustained antidepressant actions (14). Studies supporting the safety and efficacy of repeated ketamine dosing are emerging (15). Antagonism of NMDA-type glutamate receptors in the central nervous system may be a possible mechanism for ketamine’s action in catatonia (16).

To our knowledge, there are only two other case reports documenting the use of ketamine to treat catatonia (12). The response was rapid and complete; however, the underlying cause of catatonia was unclear. The two cases discussed in this report were both bipolar in nature and it remains unclear how catatonias of alternative underlying pathology would respond. Given that ketamine can induce or exacerbate symptoms of psychosis in healthy and schizophrenic participants (17, 18), these findings do not provide evidence of the efficacy or safety of ketamine in catatonia secondary to other conditions, such as catatonic schizophrenia. Both presentations were complicated by multiple medical co-morbidities. The number of treatments required varied between the patients, however, both showed a significant response within the first few treatments. The treatment was well tolerated by the first patient, and the second patient requested to stop treatments due to dissociative side-effects.

These cases indicate ketamine is worthy of further investigation as a rapidly acting treatment for catatonia in bipolar depression, especially given the increased risks associated with ECT and limited access in some areas during the COVID-19 pandemic.

## Data availability statement

The raw data supporting the conclusions of this article will be made available by the authors upon email request.

## Ethics statement

The studies involving human participants were reviewed and approved by the Gold Coast Hospital Human Research Ethics Committee. The patients/participants provided their written informed consent to participate in this study. Written informed consent was obtained from the participants for the publication of any potentially identifiable images or data used in this article.

## Author contributions

SS conceptualized the case report and reviewed the manuscript. GB managed the literature searches and analyses. MK undertook the statistical analysis. AA wrote the first draft of the manuscript. All authors contributed to the article and approved the submitted version.
